# Does urbanisation have an impact on the trophic ecology of the Algerian hedgehog *Atelerixalgirus* in northern Algeria?

**DOI:** 10.3897/BDJ.12.e115721

**Published:** 2024-01-29

**Authors:** Amina Zidane, Ilham Sahki-Benabbas, Mohamed Ayoub Rahal, Riadh Moulaî

**Affiliations:** 1 Laboratory of Dynamics and Biodiversity, Faculty of Biological Sciences, University of Science and Technology Houari Boumediene, LP 32 El Alia, Bab Ezzouar, Algiers, Algeria Laboratory of Dynamics and Biodiversity, Faculty of Biological Sciences, University of Science and Technology Houari Boumediene, LP 32 El Alia, Bab Ezzouar Algiers Algeria; 2 Laboratory of Applied Zoology and Animal Ecophysiology, Faculty of Biological sciences, University of Bejaia, Bejaia, Algeria Laboratory of Applied Zoology and Animal Ecophysiology, Faculty of Biological sciences, University of Bejaia Bejaia Algeria

**Keywords:** hedgehog, northern Algeria, urbanisation, trophic-ecology

## Abstract

The Algerian hedgehog, which is an endemic Mediterranean species, is a nocturnal and terrestrial insectivorous mammal. *Atelerixalgirus*’ populations are widespread in various habitats comprising human agglomeration, such as rural, suburban or natural ecosystems. However, the impact of the habitat’s characteristics on its diet remains unknown in Algeria. To contribute to a better understanding of this question, we have analysed 158 faeces samples of the Algerian hedgehog in three different areas: urban, suburban and natural area.

The findings show that the Algerian hedgehog is an opportunistic species. It feeds on several classes of Arthropoda, but the harvester ant *Messorbarbarus* dominates largely its menu with AR = 74.81% in the diverse habitats. Furthermore, the HAC and FCA analyses confirm the positive impact of the level of urbanisation and the anthropogenic activity on the Algerian hedgehog prey richness in the north of Algeria (79 prey species and 5574 individuals ingested in the urban area, compared to 64 prey species and 3188 individuals ingested in the natural zone).

## Introduction

Human influences on ecosystems have kept increasing in the last decades which is noticeable in the several changes made in their environment to meet their needs, especially for their food (agricultural activities and animal breeding) and habitat (infrastructure, transport pathways and means of communication). These changes and transformations have detrimental and irreversible consequences on biodiversity and the ecosystem functioning (Mckinney 2006, Ellis and Ramankutty 2008) as they cause habitat fragmentation for several species.

Hedgehogs are small, nocturnal and spiny-haired mammals found in synanthropic environments (Jarosz et al. 2010, Silaghi et al. 2012, Kryštufek and Motokawa 2018, Andreychev and Kuznetsov 2020). Recently, they have become popular amongst pet owners due to their potential benefits as they feed on insects, worms and other small harmful animals to vegetation. The International Union for Conservation of Nature (IUCN) evaluated these animals as not being a focus of species conservation (the least concern category) (Amori et al. 2008). The IUCN estimated that the number of hedgehogs is decreasing because of several factors impacting their populations' density worldwide. For example: road accidents as reported by Mouhoub-Sayah et al. (2009) in the Soummam Valley in Algeria, diseases, hunting and predation. However, their nocturnal behaviour makes it hard to identify the fluctuations in its numbers.

In order to protect animals, the trophic ecology is the most important factor to study. In Algeria, studies concerning the diet of *Atelerixalgirus* are numerous like those carried out on the Highlands near the Boughzoul Dam (Baaziz 1991), in the Mitidja area (Doumandji and Doumandji 1992), in the Great Kabylie (Sayah 1988, Bendjoudi and Doumandji 1996, Mimoun and Doumandji 2006, Brahmi et al. 2012) and near the marsh of Reghaia (Biche 2003, Baouane 2005, Ouarab and Doumandji 2010) in the Merguebe Reserve.

The purpose of our study is to understand the dietary habits of hedgehogs in areas with varying degrees of anthropogenisation. The main objective is to analyse how human influence on the environment can impact the feeding behaviour of these animals. By selecting data collection stations in diverse environments, ranging from densely urbanised areas to those less frequented by humans (natural areas), the study aims to identify the dietary preferences of hedgehogs, based on their habitat altered by human activity. This deeper understanding can provide crucial information for species conservation and the management of urban spaces to promote harmonious co-existence between hedgehogs and human activities.

## Material and methods

To conduct this study, the sampling process was conducted over the period spanning from March 2017 to October 2018. The study focused on three distinct areas, each exhibiting different levels of urbanisation, all situated in the northern part of Algeria.

### Study area

**Figure 1.**
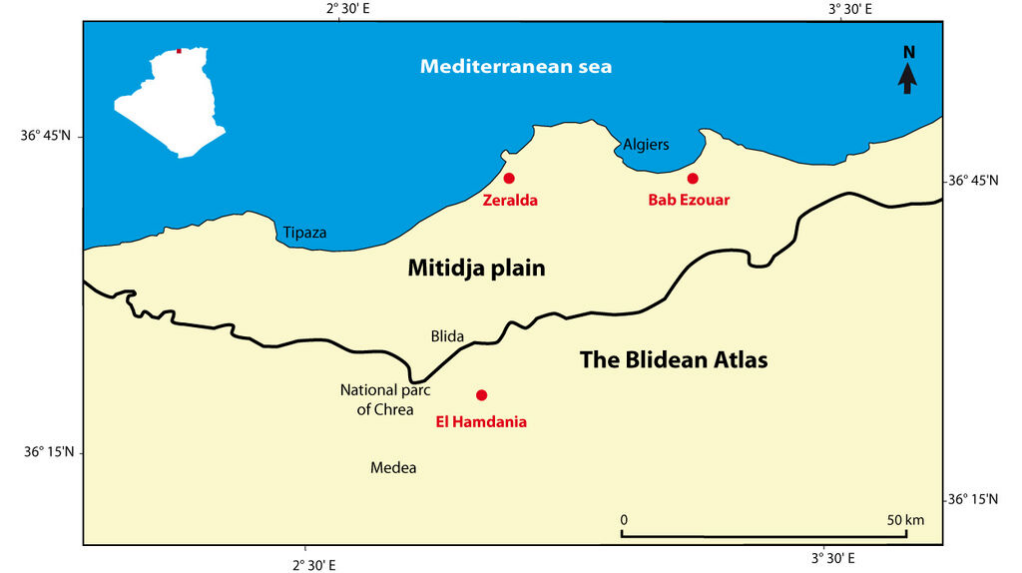
Geographical location of the three study areas in northern Algeria.

Three study areas have been selected to undertake this study in northern Algeria (Fig. 1), where two are part of the Mitidja Plain, considered the largest sublittoral plain in Algeria according to Mutin (1977). The choice of study areas is primarily based on the presence of a sufficient number of accessible droppings for collection. Subsequently, the selection is made according to the degree of urbanisation and the intensity of anthropogenic activity. The first one (Bab Ezouar) is close to housing and is subject to a strong anthropogenic influence, the second (Zeralda) is located in a suburban area, while the third site (El Hamdania) is a forest type (natural environment).

#### Urbanised area

**Figure 2.**
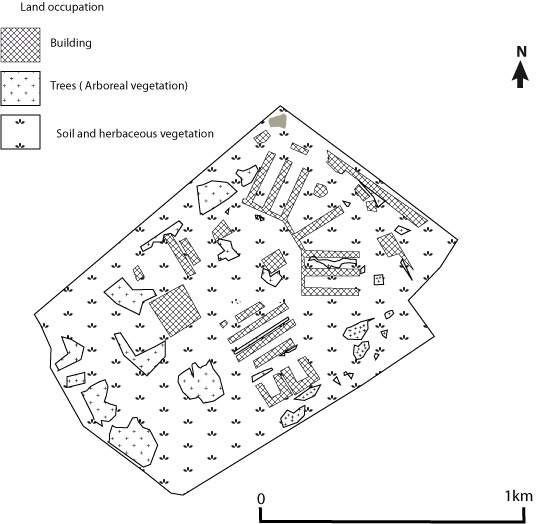
Map of land occupation in the urbanised area (Bab Ezouar).

This area is located in Bab Ezouar within the campus of the University of Science and Technology Houari Boumediene (USTHB) (Fig. 2). It extends over former cereal and forage farmland near the Algiers airport, covering 105 ha of which 45 ha is open space. This area was partially swampy along the railway line. Currently, with the installation of the campus, this zone has seen an important reorganisation of the land, the topography and the vegetation which makes it a favourable environment for both the fauna and the flora.

#### Suburban area

**Figure 3.**
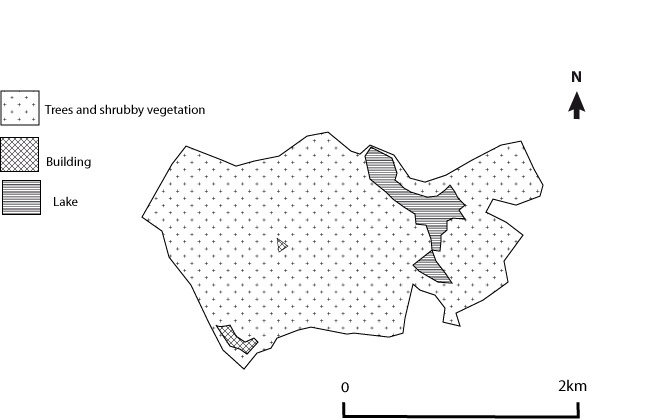
Map of land occupation in the suburban area (Zeralda).

This sampling station is located in a coastal region within the hunting centre of Zeralda (Fig. 3). It covers an area of 19.8 ha and is situated 30 km west of Algiers. This station is limited to the north-east and to the south by the hunting reserve of Zeralda and the west by the department road number 13 connecting Zeralda to the commune of Mahelma. It corresponds to an old arboretum.

The two sampling stations of the Mitidja Plain present a Mediterranean climate (Table 1) with two main periods: one rather rainy and cool lasting from late autumn to early spring. The other one is hot and dry lasting four months.

#### Natural area

**Figure 4.**
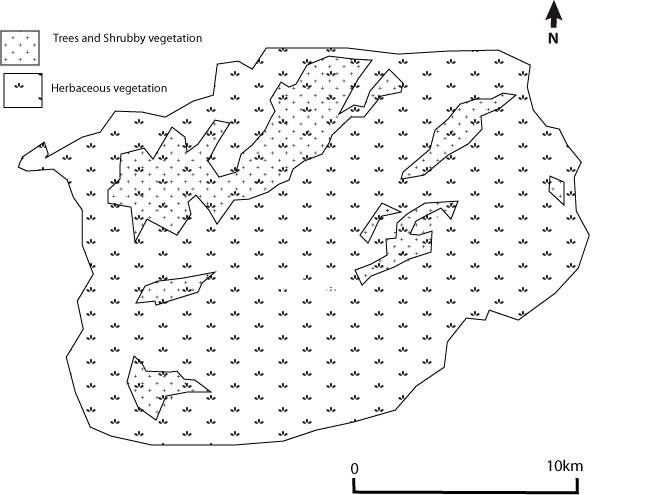
Map of land occupation in the natural area (El Hamdania).

The site of El Hamdania is located in the western zone of the National Park of Chrea. Its surface is 8825 ha and situated at an altitude of 800 m (Table 1). It is limited by Djbel Msenou to the north, Beni Mesoud to the east, El Hamdania to the west and Zondai to the south. This area was chosen for its isolation from any anthropogenic activity (Fig. 4). It belongs to the bioclimatic stage: sub-humid with a cool winter.

### Methodology

The faeces identification is relatively simple and the risks of confusion with other mammals’ faeces are very low. Faecal samples were collected from March 2017 to October 2018, early in the morning then transported to the laboratory where they were marked (date and place of collection) after weighing and taking measurements. The analyses consisted of identifying and counting the remains of undigested prey contained in the droppings. Using a binocular magnifier, the sclerotinised pieces were separated and grouped according to their similarities, then we proceeded to the identification of each grouping. Prey fragments are determined largely by reference to the collections held in the applied zoology laboratory at the University of Bejaia (Algeria), but also thanks to a number of identification guides and specialised websites (Jones et al. 1983, Zahradnik 1984, Gaêtan 1986, Patrice and Philippe 2003, Louveaux et al. 2013). For each study site, we established the faunistic list of prey taxa consumed according to their abundances.

### Data analysis

During the sampling period, a total of 158 faecal samples were collected, distributed across different environments. Of these, 65 samples were obtained from the urban area, 48 from natural area and 45 from suburban area.

For the examination of potential variations in the diets of *Atelerixalgirus*, we have used the ecological composition index: the frequency of occurrence Fo %, the centesimal frequency Fc %, the Shannon diversity index (H'), the Equitability (E) and the Sorensen similarity index (Dajoz 2006, Ramade 2008). To describe the *Atelerixalgirus* diet variations amongst the three study areas, we used the Costello graphical representation (Costello 1990), modified by Amundsen et al. (1996). It highlighted the importance of prey, their contribution to the trophic niche extent and their feeding strategy (Loua et al. 2019). The Kruskall-Wallis test was applied to test the differences between the samples. It is a non-parametric test suitable when the data do not follow a normal distribution. In the context of the study of diet, the distribution of data may be influenced by the natural variability of food resources in different environments (this is the case of our study, three groups of independent samples, etc.).

To identify the relationship between the ingested prey and the ecological characteristics of each study area, such as the level of anthropogenisation, the floristic richness (depending on the vegetation strata) and the opening level of the environment, we have applied a factorial analysis of correspondences (FAC). The results of this analysis were confirmed by the hierarchical ascending classification (HAC) which used the Euclidian distance between the variables. The test was done by using the Software XLSTAT 2021.

## Results

### Diet variation

**Figure 5.**
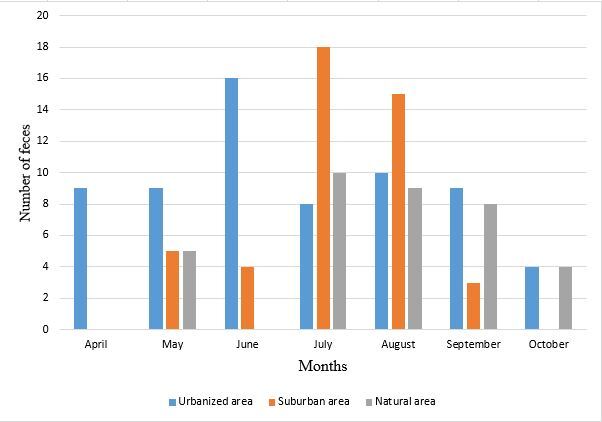
Monthly abundance of droppings collected in the three study sites.

The highest abundance of faeces was recorded in the urbanised area with 65 faecal droppings between April and October. We noticed a total absence of droppings during April and October in the suburban area and during April in the natural area (Fig. 5). The number of droppings collected was constantly increasing from April to August, but seems to decrease from September. However, from November to March, we have not observed any afecal dropping of *Atelerixalgirus* in the three study sites.

**Figure 6.**
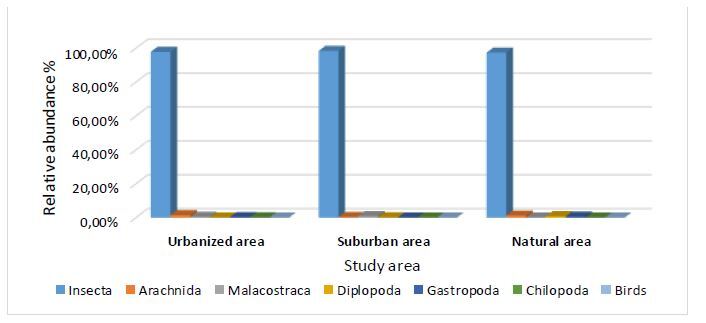
Frequency of prey taxa consumed by *A.algirus* in the three study sites in Algeria.

One hundred and sixty-one prey species were found in the Algerian hedgehog diet. The prey taxa were divided into seven classes, in which the class Insecta is the dominant and represented 97.72 % of the consumed prey (Fig. 6).

The list of prey ingested by the Algerian hedgehog in the three study sites can be found in Table 2.

Amongst the Insecta class, the Hymenoptera order is the most consumed (Hymenoptera AR = 86.90%) in the three study areas. This order is mainly represented by the Formicidae family. The species *Messorbarbarus* is the most consumed prey; its frequencies were 83.36% in the urbanised habitat (Bab Ezouar), 77.62% in the sub-urbanised habitat (Zeralda) and 67.25% in the natural habitat (El Hamdania).

The order of Coleoptera (AR = 8.123%) took second place, mainly represented by the two families: Carabidae and Tenebrionidae. The ant *Camponotus* sp. 1 came in the second position after *Messorbarbarus* with Fc = 22.15% in the suburban area and Fc = 12.81% in the natural area.

As for the urban area, the second favourite prey consumed by *A.algirus* was the Carabidae *Acinopuspicipes* with Fc = 2.88%.

The third position is attributed to different species in the three study sites: the Blattelidae *Ectobius* sp. in the urban area with Fc = 1.14%, the Carabidae beetle *Calathus* sp. with Fc = 2.75% in the sub-urban area and *Phyllodromicaalgirica* with Fc = 4.62% in the natural area.

The findings show that the diversity and abundance of prey consumed are the highest in the urban area (Table 3) where we have recorded the presence of 79 prey species divided over 5574 individuals.

The parameters decreased in the natural area with 64 species and 3188 individuals. The suburban environment, on the other hand, had the lowest diversity and abundance (45 species and 1994 individuals consumed).

The difference between the samples were non-significant and confirmed by the Kruskall- Wallis test where: H (chi2): 18.24, p > 0.05.

The determination of the amplitude of the trophic niche in the three study areas showed no significant difference in the diversity and equitability index. The values of equitability tend to zero in the different types of environments (Table 3) which determine a situation of imbalance between the consumed prey and that only one species dominated the diet of *A.algirus* in the three environments and, thus, represented nearly the totality of the population (heterogeneous population).

The Sorensen's similarity index showed a slight similarity between the three environments (Table 4): urban and suburban areas seemed to share more similarity of consumed prey Is = 0.64; the natural area, on the other hand, showed little similarity of ingested prey (0.38 with the urban area and 0.35 with the sub-urban area).

**Figure 7.**
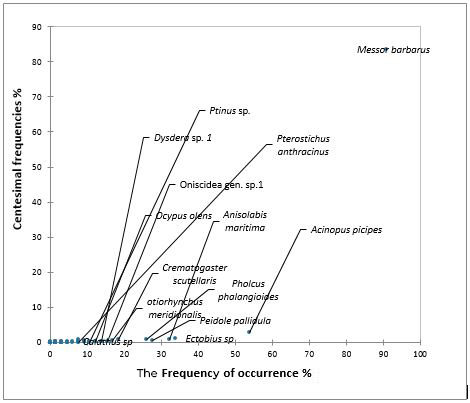
Prey species ingested by *Atelerixalgirus* in the urban area in northern Algeria.

The Costello graphical representation described the Algerian hedgehog as a generalist predator (feeding on a wide variety of prey) and inhabiting a broad ecological niche in all three environments. The ant *Messorbarbarus* is qualified as the dominant prey. In the urban areas, the three types of prey, known as secondary, are represented by *Acinopuspicipes*, *Anisolabismaritima* and *Tetramoriumbiskrensis* (Fig. 7).

**Figure 8.**
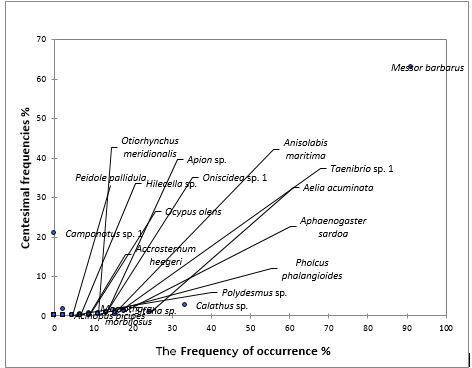
Prey species ingested by *Atelerixalgirus* in the suburban area in northern Algeria.

In the suburban area (Fig. 8), they were represented by *Camponotus* sp. 1, *Othiorynchusmeridionalis*, *Sehirus* sp., *Aeliaacuminata* and *Aphaenogastersardoa*.

**Figure 9.**
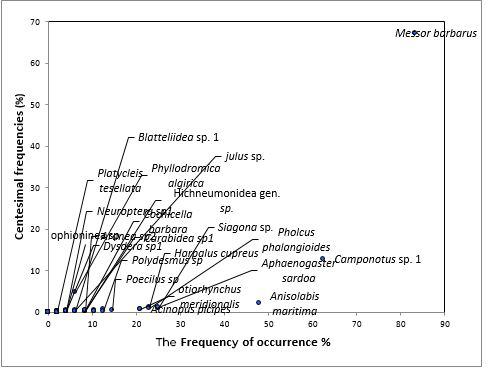
Prey species ingested by *Atelerixalgirus* in the natural area in northern Algeria.

In the natural area, the species *Anisolabismaritima* and *Crematogasterscutellaris* were secondary prey (Fig. 9). According to this representation, the other prey consumed are qualified as rare species or accidentally ingested by the Algerian hedgehog.

### Impact of anthropogenisation on Atelerixalgirus’s diet

**Figure 10.**
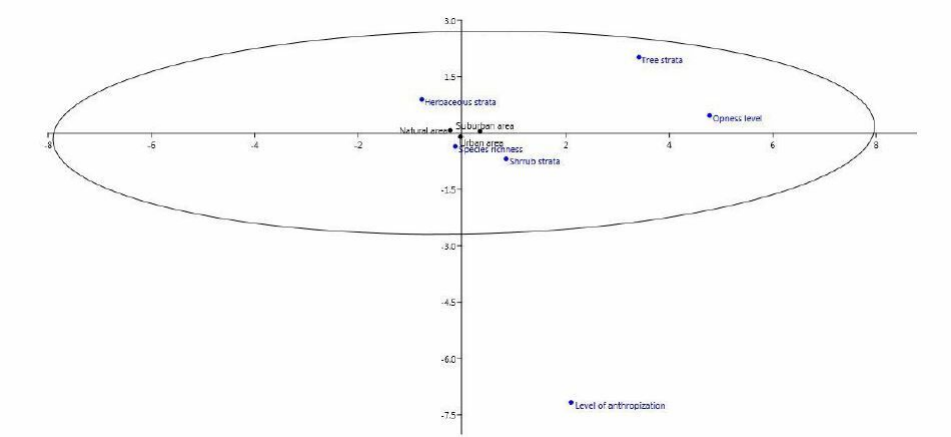
Correspondence Factorial Analysis considering the study sites and the characteristic variables relating to the diet of the Algerian hedgehog.

In the interests of evaluating the effect of biotic factors (level of anthropogenisation, number of floral taxa in the environment according to strata and openness level of the environment) on the richness of prey items consumed, we applied a factorial correspondence analysis (Fig. 10).

According to Axis F1 = 87.03%, we observed in the three study areas that the number of prey consumed depends on the density and composition of the vegetation cover particularly on the cover of the herbaceous and shrub.

The type of vegetation cover predominating the area, conditions directly the environment's degree of openness. The environment is open when the herbaceous strata is dominant (according to the F2 = 12.96%) and closed if trees are dominant.

The number of prey items consumed by hedgehogs does not depend on the anthropogenisation level, so hedgehogs adapted the same feeding strategy in different types of environments (Fig. 10).

**Figure 11.**
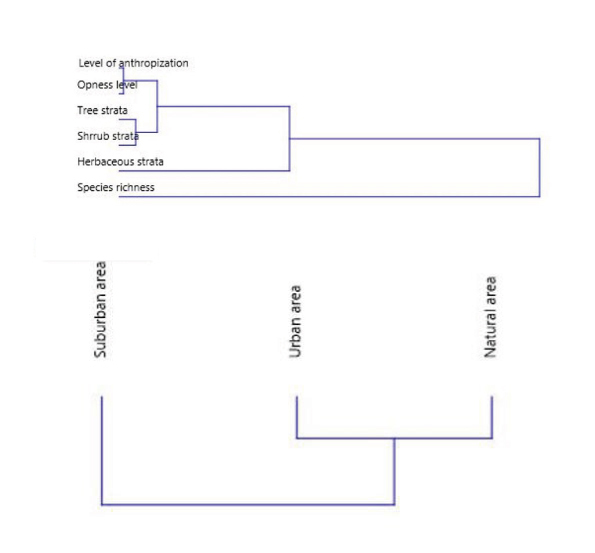
Ascending hierarchical classification of habitat characteristic variables and study sites relating to the diet of the Algerian hedgehog.

The findings of the FCA are confirmed by the ascending hierarchical classification using the Euclidean distance to classify the variables (Fig. 11) where we observed two major groupings: the first is formed by the consumed prey and the herbaceous strata. The second is formed by the openness level and the anthropogenisation level, these results confirming those previously obtained by the FCA.

With the aim of grouping the study sites, we observed a first grouping formed by the natural area and the urban area according to affinity (they share more similarities). The suburban zone was distant from this group.

As for the variables, the number of prey consumed depends on the percentage of the herbaceous strata in the area (according to the distance between them). The openness level presented less affinity with the previous variables, which confirmed the results of the FCA.

## Discussion

### Diet composition

The collection of Algerian hedgehog faeces showed that this species frequented the three different areas despite their heterogeneity (urban, suburban and natural environment). Reeve (1994) also reported that the European hedgehog (*Erinaceuseuropaeus*) tends to use a wider range of habitats ranging from forest to humanised environments.

The urban area (at 14 m altitude) was the most frequented (65 faeces samples) during the months of sampling compared to the other study sites (suburban and natural). Doumandji and Doumandji (1992) pointed out that the hedgehog does not hibernate in the coastal area at less than 50 m. However, it tends to reduce its activity, which explains the presence of droppings between April and October and their absence from November to March, because of the reduced activity in urban areas or hibernation in the other two stations (suburban: 100 m and natural: 800 m). Mouhoub-Sayah et al. (2018) recorded the highest faeces number during the pre-hibernation period because it represents a period of body reserve accumulation for hibernation.

The findings show that the Algerian hedgehog not only feeds on Insecta (97.72%), but also on Arachnida, Malacostraca, Diplopoda, Gastropoda, Chilopoda and Birds. This is confirmed by several studies (Schilling et al. 1986, Doumandji and Doumandji 1992, Mouhoub-Sayah et al. 2009, Khaldi 2014). The class of Birds recorded a single individual ingested corresponding to an egg. Axell (1956) and Boue and Chanton (1967)confirmed that *A.algirus* can also consume young birds and eggs on the ground.

The most consumed order of Insecta in the three study sites is the Hymenoptera with 86.90%, essentially represented by the Formicidae family.

The ant *Messorbarbarus* is the most consumed prey by *A.algirus* in the three study sites with AR = 87%. This species is qualified according to the Costello diagram as the dominant prey in the diet of the hedgehog (AR > 50%).

The Kruskall-Wallis test applied to identify the difference in prey between samples was found to be insignificant which confirms that, despite the difference in habitats, *A.algirus* still follows the same strategy as a generalist predator with an opportunistic character feeding on *Messorbarbarus*. This corresponds with the results of Mouhoub-Sayah et al. (2009) in the Soummam Valley in Algeria.

The Equitability index confirmed those data because it has a low value in the three study sites, which reflected a situation of imbalance (heterogeneous population) to the benefit of *M.barbarus*. Derdoukh et al. (2012) considered that the ant *Messorbarbarus* is the most dominant prey in the diet of *Atelerixalgirus* in two stations located in the Mitidja Plain.

The Coleoptera order is the second most consumed order (AR = 8.12%), represented by *Acinopuspicipes* in the urban area. It has also been reported that the Algerian hedgehog consumed mainly Formicidae and Coleoptera, especially Carabidae (Athmani 1988, Sayah 1988). Djennoune (2019) reported that Coleoptera is the second most consumed order by *A.algirus* in three localities in Kabily, although it is quite uncommon. Our results support those reported in other studies conducted in Algeria (Doumandji and Doumandji 1992, Mouhoub-Sayah et al. 2009, Khaldi 2014). In contrast, most studies on the European hedgehog's diet (*Erinaceuseuropaeus*) have found that beetles were dominant in its faeces (Jones et al. 2005, Jones and Norbury 2011) in New Zealand.

### Impact of anthropogenisation on Atelerixalgirus' diet

The highest prey richness was recorded in the urban area (79 species). This sampling station is characterised by a mosaic landscape composed of the university’s campus buildings and open spaces where the herbaceous strata dominates (Fig. 2Table 1). Our results corroborate those of Hubert et al. (2011) who state that urban or peri-urban areas harbour hedgehogs in large numbers, about nine times more than in non-urban areas. Khidas (1998) has also noted the presence of *A.algirus* faeces in various open habitats in Algeria. In the natural area, in a national park, we also recorded 64 prey species of the Algerian hedgehog. This site presented a low anthropogenic activity; it is far from all types of human constructions. It is located at an altitude of 800 m; furthermore, it is characterised by a mountainous relief and a dense vegetation cover dominated by the herbaceous strata (Fig. 4Table 1). Djennoune (2019) also reported the presence of *A.algirus* faeces in Yakouren, which is a mountainous station located between 280 m and 1340 m altitude. Hubert (2008) revealed that stations located at high altitude with mountainous relief correspond to the availability of the hedgehogs' preferred natural food (insects mainly ants).

Reeve (1994) suggested that the hedgehog habitat depends on the existence of shelters (natural hedges and bushes), which are available in semi-open environments, such as those of El Hamdania and the university campus in urban areas.

The suburban area recorded the lowest number of faeces (45 faeces samples) and the lowest prey richness (45 species). Despite the reduced anthropogenic effect compared to the urban area, this site is characterised by the domination of two shrub and tree strata which make the habitat less open (Fig. 3). This vegetation cover harbours a few prey items that interest the hedgehog as it feeds on Arthropods that are found on the ground (depending on its shifting mode). Garcia and Puig (2014) concluded that the optimal biotope for the hedgehog is the open and semi-open environment with abundant herbaceous and shrub strata, this being confirmed in Algeria by the present study and the one of Djennoune (2019).

The presence of the hedgehog in the three different areas confirms that it uses all available habitat types. This is related to its predation strategy, which qualifies *Atelerixalgirus* as an opportunistic generalist. Although it has food preferences, we noted, through the results of the FCA and HAC, that the richness of its prey is directly related to the dominance of the herbaceous strata in the first place because it harbours the hedgehogs' prey species.

The openness level of the environment joined the grouping in the second place because it results from the type of strata that dominates each environment. The urban environment is "open", the suburban environment and the natural environment being of semi-open type. According to literature, these two levels of openness are responsible for the food availability of the hedgehog (Garcia and Puig 2014).

The urban area, which presents a great anthropogenic activity, recorded the highest richness of prey and an important abundance of faeces than the other habitat types. It indicated that the level of anthropogenisation does not condition the activity of the Algerian hedgehog. This is confirmed by the FCA's results where the level of anthropogenisation does not belong to the grouping obtained and is also confirmed by the HAC where it comes in last place to join the parameters influencing *Atelerixalgirus* prey’s richness.

The human activities in those areas do not have a strong influence on hedgehog populations. This is confirmed by the studies of Djennoune (2019) in Algeria. Anthropogenic activity and urbanisation do not seem to negatively affect the activity and diet of the Algerian hedgehog; instead, the urban environments appear to be conducive to hedgehogs due to higher food densities related to human occupation including natural prey, anthropogenic sources, more suitable breeding sites and reduced predation risk (Morris 1985, [Bibr B10564296][Bibr B10564477], Pettet et al. 2017).

## Conclusions

In conclusion, we can say that the Algerian hedgehog confirms its status as a generalist terrestrial predator. It feeds on several classes, encompassing a variety of prey types. Notably, the Insect class stands out as the most abundant, with a particular emphasis on the ant species *Messorbarbarus*. These results highlight the pronounced preference for *Messorbarbarus* as a primary food source for the hedgehog across all three study areas.

The hedgehog exhibits a remarkable capacity for adaptation across the three distinct study areas, showcasing its ability to thrive in varied environmental conditions.

This species is highly influenced by both the type of vegetation and its physionomy, but it was little influenced by the anthropogenisation level of habitats.

Open and semi-open environments with short herbaceous vegetation, a few scattered trees and shrubs are the preferred feeding habitats for hedgehogs. Urban areas, if they have open spaces with vegetation and shelters, may be more favourable for food search and the availability of prey.

Overall, the hedgehog's adaptability across the three study areas is a testament to its ecological versatility, allowing it to exploit diverse resources and exhibit behavioural flexibility in response to the varying degrees of anthropogenic influence and habitat alteration. Urbanisation does not appear to have a detrimental impact on the Algerian hedgehog. On the contrary, the species has demonstrated a successful adaptation to human presence, underscoring the imperative to preserve it.

In perspective, it would be interesting to study the place of the Algerian hedgehog in food chains in environments presenting different degrees of anthropogenisation to precisely characterise its feeding habitat to better conserve this endangered species.

## Figures and Tables

**Table 1.** Summary of important features of the three study areas.

**Area type**	**Urban area**	**Suburban area**	**Natural area**
**Geographic situation**	Mitidja Plain	Mitidja Plain	Blidean Atlas
**Latitude, Longitude**	36°42'49.13"N 3°10'52"E	36°42′06″N 2°51′47″E	36°21′25.55"N 2°48'3.86"E
**Altitude**, **m**	4	100	800
**Surface area**, **ha**	105	19.75	400
**Bioclimatic Stage**	Sub-humid with mild winter	Sub-humid with mild winter	Sub-humid with cool winter
% **of the vegetation**	67	70	82
**Dominant stratum**	Herbacuous	Shrubby/trees	Herbaceous
**Dominant Species**	*Arisariumvulgare*, *Avenasterilis*	*Oleaeuropea*, *Quecussuber*	*Genistatricuspidata*, *Trifoliumglomeratum*, *Festucaatlantica*
**Level of anthropogenisation**	High	Medium	Very low

**Table 2.** Prey species ingested by the Algerian hedgehog *Atelerixalgirus* at three stations in northern Algeria.

**Class**	**Order**	**Family**	**Species**	**N Bab Ezouar**	**N Zeralda**	**N El Hamdania**
Birds			Sp. ind.	0	0	1
Chilopoda	Lithobiomorpha	Lithobiidae	*Lithobius* sp.	6	0	0
Gastropoda	Stylomatophora	Helicidae	*Helix* sp.	0	1	0
		Geomitridae	*Cochicellabarbara* (Linnaeus, 1758)	3	0	12
	Pulmonata	Hygromiidae	*Hilecella* sp.	5	4	6
		Polydesmidae	*Polydesmus* sp.	3	2	9
Diplopoda	Julida	Julidae	*Julus* sp.	0	0	14
Malacostraca	Isopoda	Oniscidae	Oniscidae gen. sp. 1	20	18	2
			Oniscidae gen. sp. 2	3	0	0
Arachnida	Scorpiones	Buthidae	*Buthusoccitanus* (Amoreux, 1789)	0	0	3
	Araneae	Dysderidae	*Dysdera* sp.	13	0	4
		Araneidae	*Araneus* sp. 1	8	0	2
			*Araneus* sp. 2	2	0	6
		Gnaphosidae	Gnaphosidae gen. sp.	11	0	0
		Pholcidae	*Pholcusphalangioides* (Fûssli, 1775)	45	7	25
		Oribatida	Oribatida gen. sp.	1	0	0
Insecta	Orthoptera		Orthoptera gen. sp.	0	0	1
			*Tessellanatesellata* (Charpentier, 1825)	0	0	3
		Gryllidae	*Gryllus* sp. 1	1	0	0
			*Gryllus* sp. 2	9	0	0
		Acrididae	*Anacridium* sp.	0	3	0
	Blattodea	Blattidae	Blattidae gen. sp.	3	2	0
		Blattellidae	Blattellidae gen. sp.	8	0	3
		Ectobiidae	*Ectobius* sp.	64	1	7
			*Phyllodromicaalgerica* (Bolívar, 1881)	19	0	148
	Neuroptera	Myrmeleontidae	Myrmeleontidae gen. sp. 1	0	0	2
			Myrmeleontidae gen. sp. 2	9	2	10
	Diptera	Stratiomydae	Stratiomyidae gen. sp. 1	1	0	0
			Stratiomydae gen. sp. 2	1	0	0
	Hemiptera	Pentatomidae	*Aeliaacuminata* (Linnaeus, 1758)	5	18	7
			*Accrosternumheegeri* (Fieber, 1861)	1	6	0
		Cydnidae	*Sehirus* sp.	12	1	0
	Dermaptera	Anisolabididae	*Anisolabismaritima* (Bonelli, 1832)	54	16	65
			*Carcinophoria* sp.	4	0	0
			Dermaptera gen. sp.	0	0	1
	Coleoptera	Cerambycidae	*Cerambyx* sp.	0	0	1
		Elateridae	Elateridae gen. sp.	1	1	1
		Chrysomelidae	*Timarcha* sp.	0	0	3
		Scarabaeidae	*Mimela* sp.	1	0	0
			*Ontophagus* sp.	1	0	0
			*Onitis* sp.	0	5	3
			*Rhizotrogus* sp.	0	0	1
			Scarabaeidae gen. sp. 1	0	1	1
			Scarabaeidae gen. sp. 2	0	0	1
			*Scarabaeus* sp.	0	1	0
		Apionidae	*Apion* sp.	2	15	0
		Ptinidae	*Ptinus* sp.	7	0	0
		Staphylinidae	*Astenu* sp.	9	0	1
			*Ocypusolens* (O.F. Mûller, 1764)	16	5	19
			Staphylinidae gen. sp.	1	0	0
		Curculionidae	Curculionidae gen. sp. 1	1	0	0
			Curculionidae gen. sp. 2	0	0	1
			Curculionidae gen. sp. 3	4	0	0
			*Hypera* sp.	19	7	0
			*Otiorhynchusmeridionalis* (Gyllenhal, 1834)	29	12	24
			*Sitona* sp.	3	26	0
		Tenebrionidae	*Alphasidagrossa* (Solier, 1836)	0	3	0
			*Asida* sp.	19	1	0
			*Bioplanesmeridionalis* (Mulsant, 1854)	0	3	0
			*Blaps gibba* (Laporte De Castelnau, 1840)	0	0	2
			*Cryphaeus* sp.	0	1	0
			*Dendaruscoarcticollis* (Mulssant, 1854)	7	0	0
			*Erodius* sp.	0	2	8
			*Micrositus* sp.	1	0	0
			*Nalassus* sp.	0	32	0
			*Opatrum* sp.	0	0	1
			*Pachychilaservillei* (Sollier, 1835)	0	0	6
			*Pimelia* sp.	0	15	6
			*Scaurus* sp.	19	1	0
			*Stenosis* sp.	1	0	0
			*Taenibrio* sp. 1	5	7	5
			*Taenibrio* sp. 2	20	1	0
		Carabidae	*Acinopuspicipes* (Olivier, 1795)	161	1	23
			*Calathus* sp.	7	55	0
			Carabidae gen. sp. 1	1	1	12
			Carabidae gen. sp. 2	1	0	0
			*Carterus* sp.	0	0	14
			*Chlaenius* sp.	1	0	0
			*Carabusalysidotus* (Illiger, 1798)	0	0	1
			*Carabus* sp.	2	1	1
			*Ditomuscalydonius* (P. Rossi, 1790)	0	0	1
			*Ditomus* sp.	3	0	1
			*Dromius* sp.	3	0	3
			*Harpaluscupreus* (Dejean, 1829)	0	0	34
			*Harpalushonestus* (Duftschmid, 1812)	0	0	3
			*Harpalus* sp. 1	3	0	0
			*Harpalus* sp. 2	0	0	9
			*Licinussilphoides* (P. Rossi, 1790)	11	1	0
			*Macrothoraxmorbillosus* (Fabricius, 1792)	3	4	0
			*Molops* sp.	1	0	0
			*Percus* sp.	2	0	2
			*Poecilus* sp.	0	2	13
			*Pterostichusanthracinus* (Illiger, 1798)	5	0	4
			*Scarites* sp.	3	0	4
			*Siagona* sp.	13	0	25
	Hymenoptera	Vespidae	Vespidae gen. sp.	1	0	0
		Dermestidae	*Anthrenus* sp.	1	0	0
		Chrysomelidae	*Chrysolina* sp.	0	2	0
		Ichneumonidae	Ichneumonidae gen. sp.	0	0	4
			Ophioninae gen. sp.	7	0	6
		Formicidae	*Tapinoma* sp.	1	0	0
			*Camponotus* sp. 2	0	0	2
			*Monomorium* sp.	3	0	0
			*Plagiolepis* sp.	5	0	0
			*Tapinomanigerrimum* (Nylander, 1856)	6	0	0
			*Cataglyphisviaticus* (Fabricius, 1787)	7	0	1
			*Tetramoriumbeskrensis* (Forel, 1904)	9	0	0
			*Ctaglyphisbicolor* (Fabricius, 1793)	10	0	0
			*Crematogaster* sp. 1	13	4	8
			*Messor* sp. 1	40	0	0
			*Pheidolepallidula* (Nylander, 1849)	33	5	8
			*Crematogasterscutellaris* (Olivier, 1792)	52	0	0
			*Aphaenogastersardoa* (Mayr, 1853)	33	23	39
			*Camponotus* sp. 1	9	419	409
			*Messorbarbarus* (Linnaeus, 1767)	4647	1256	2144
			Hymenoptera gen. sp.	0	0	2

**Table 3.** Different ecological indices for prey consumed by *A.algirus*. (H'= Shanon-Weaver index, H_max_= maximum diversity, E= Equitability).

**Parameters /Area**/	**Urban**	**Suburban**	**Natural**
**Faeces number**	65	45	48
**Total richness**	79	45	64
**Prey abundance**	5574	1994	3188
**H’ (bits)**	1.037	1.404	1.49
**H_max_ (bits)**	6.30	5.45	6
**E**	0.23	0.36	0.35

**Table 4.** Sorensen's Similarity Index amongst the three study sites.

	**Urban area**	**Suburban area**	**Natural area**
**Urban area**	0	0.64	0.38
**Suburban area**	0.64	0	0.35
**Natural area**	0.38	0.35	0
